# Bovine leukemia virus p24 antibodies reflect blood proviral load

**DOI:** 10.1186/1746-6148-8-187

**Published:** 2012-10-09

**Authors:** Gerónimo Gutiérrez, Hugo Carignano, Irene Alvarez, Cecilia Martínez, Natalia Porta, Romina Politzki, Mariela Gammella, Marina Lomonaco, Norberto Fondevila, Mario Poli, Karina Trono

**Affiliations:** 1 Instituto de Virología. Centro de Investigaciones en Ciencias Veterinarias y Agronómicas, INTA, C.C. 1712, Castelar, Argentina; 2 Instituto de Genética. Centro de Investigaciones en Ciencias Veterinarias y Agronómicas, INTA, C.C. 1712, Castelar, Argentina

**Keywords:** BLV, Proviral load, Control measures, Serological marker, ROC curve, p24 antibodies

## Abstract

**Background:**

Bovine leukemia virus (BLV) is worldwide distributed and highly endemic in Argentina. Among the strategies to prevent BLV dissemination, a control plan based on the selective segregation of animals according to their proviral load (PVL) is promising for our dairy productive system. The objective of this work was to study the relationship between the blood PVL and the antibody level, in order to identify whether the individual humoral response, i.e. the anti-p24 or anti-whole-BLV particle, could be used as a marker of the blood level of infection and thus help to recruit animals that may pose a lower risk of dissemination under natural conditions.

**Results:**

The prevalence of p24 antibodies on the 15 farms studied was over 66%. The prevalence of p24 and whole-BLV antibodies and PVL quantification were analyzed in all the samples (n = 196) taken from herds T1 and 51. ROC analysis showed a higher AUC for p24 antibodies than whole-BLV antibodies (Z_reactivity_: 3.55, *P* < 0.001; Z_titer_: 2.88, *P* < 0.01), and as consequence a better performance to predict the proviral load status in herd 51. No significant differences were found between the performance of p24 and whole-BLV antibodies in herd T1. A significant positive correlation was observed between PVL values and p24 antibody reactivity in both farms (r _T1_ = 0.7, *P* < 0.001, r _51_ = 0.71, *P* < 0.0001). The analysis was extended to the whole number of weak p24 antibody reactors (n = 311) of the other 13 farms. The mean of high PVL reactors within weak p24 reactors was 17.38% (SD = 8.92). In 5/15 farms, the number of weak p24 reactors with high PVL was lower than 10%.

**Conclusions:**

We found that the humoral response reflected the level of *in vivo* infection, and may therefore have useful epidemiological applications. Whereas the quantitative evaluation of blood proviral load using real-time PCR is expensive and technically demanding, the measurement of antibodies in blood by ELISA is relatively straightforward and could therefore constitute a cost-effective tool in a BLV control intervention strategy, especially in highly infected herds such as Argentinean dairy ones.

## Background

Bovine leukemia virus (BLV), the causative agent of adult B-cell lymphosarcoma, is worldwide distributed and highly endemic in Argentina [1]. Considering the high individual prevalence on dairy farms and the absence of an official compensation policy, the application of classical control measures based on the elimination of infected cattle makes it a cost-prohibitive option. Among the strategies to prevent BLV dissemination [2], a control plan based on the selective segregation of animals according to their proviral load (PVL) is promising for our dairy productive system [3,4].

Previous studies have shown that, under experimental conditions, animals with high levels of *in vivo* infection are the most contagious ones [5]. Under field conditions, these animals could be detected and eliminated with the aim to reduce the transmission of the virus to susceptible animals. The objective of this work was to study the relationship between the blood PVL and the antibody level, in order to identify whether the individual humoral response, i.e. the anti-p24 or anti-whole-BLV particle, could be used as a marker of the blood level of infection and thus help to recruit animals that may pose a lower risk of dissemination under natural conditions.

## Methods

### Farms and samples under study

A cross-sectional study was carried out using blood samples from 15 commercial dairy farms highly infected with BLV (Table 1). One of the dairy farms raised Jersey cows (T1) whereas the remaining 14 raised Holstein cows. Samples were brought to the laboratory for BLV serology and PVL quantification because these farms are enrolled in a project that aims to analyze genetic polymorphisms related to BLV infection in the complete host genome. All lactating cattle with traceable progenitors and three or more deliveries were selected and sampled. The number of samples collected on each farm for the analysis of seroprevalence and PVL is shown in Table 1. Blood was taken by jugular venipuncture with and without heparin. Serum and whole blood was stocked frozen until analyzed. The procedures followed for extraction and handling of samples were approved by the Institutional Committee for Care and Use of Experimental Animals of the National Institute of Agricultural Technology (CICUAE-INTA) under protocol number 35/2010 and followed the guidelines described in the institutional Manual.

**Table 1:** Farms and samples under study: Seroprevalence in different herds

**Herd ID**	Animals in farm*	**Samples under study**		Seroprevalence**	Weak p24 Reactors ***	
	**n**	**n**	**%**	**%**	**%**	**Und/Low PVL (%)**
51	299	146	48.8	87.7	35.9	87.0
52	203	90	44.3	92.3	21.4	76.9
53	280	81	28.9	81.2	36.9	90.9
57	142	57	40.1	89.6	28.8	92.9
58	293	157	53.5	94.3	24.1	78.8
60	332	140	42.1	86.4	29.7	87.5
61	235	92	39.1	96.0	22.9	66.7
62	384	166	43.2	97.7	20.0	78.8
63	277	137	49.4	96.3	19.6	90.5
64	503	186	36.9	90.4	21.7	71.9
67	247	127	51.4	82.1	39.6	86.8
70	273	111	40.6	95.4	21.6	76.2
71	184	112	60.8	99.0	13.6	92.9
72	241	100	41.4	97.0	20.6	70.0
T1	250	50	20.0	66.0	27.9	91.7

*total lactating, **p24 antibodies, *** Reactivity 25-99%, % among total p24 reactors.

### BLV serology

Two in-house developed ELISA tests, using a total lysate of the BLV virus and a recombinant BLV-p24 viral core protein, designated in this study as whole-BLV ELISA and p24 ELISA, respectively, were used to detect BLV antibodies [1,6]. Normalized results were obtained as a sample to positive (S/P) ratio, and designated as reactivity. A weak positive control serum was used to calculate the ratio. Its reactivity was set to 100% and all samples were referred to it. The cut-off point was set up at 25% according to that previously described for both tests [1,6]. Depending on their reactivity, samples were stated as negative (<25), weak (25-99.99%) or strong (≥100%). The antibody titers were assayed by the end-point dilution method using two-fold dilutions of sera.

### DNA extraction and PCR amplification

Total DNA was extracted from frozen whole blood using a DNA extraction kit (High Pure PCR Template Preparation kit, Roche, Penzberg, Germany) according to the manufacturer’s instructions. The relative quantification of PVL was assessed by Taq Man real-time PCR [3]. All samples were tested in duplicate by using 50 ng of DNA as template. A fragment of the BLV *pol* gene [7] was amplified together with a fragment of the constitutive *18 S* gene [7], used as reference. As an internal control sample for both the BLV target gene and the *18 S* reference gene, we used 50 ng of DNA from fetal lamb kidney (FLK) cells, containing four copies of BLV proviral DNA per cell, in a final concentration of 1% in peripheral blood mononuclear cells (PBMCs) purified from a non-infected cow. The relative PVL was expressed as the ratio obtained by the sample for the BLV gene in comparison to the *18 S* reference gene, based on the efficiency and the cycle threshold deviation from the internal control sample [8]. With this method, the relative PVL of the control sample was set to 1 and all samples were referred to it. The reaction showed a limit of detection of 1 BLV-infected cell in 2000 non-infected cells, as previously reported [3]. The PVL was stated as undetectable if no cycle threshold value was obtained from the BLV *pol* specific reaction, low if the ratio obtained was lower than 1, and high if the ratio obtained was equal to or higher than 1. The level of BLV-infected/non-infected cells in the internal control was set up considering that the low PVL group should include only aleukemic animals, since the maximum level of provirus at this stage of infection can reach 5% of BLV-infected/non-infected cells, according to published data [9].

### Statistical analysis

The antibody levels from different PVL groups were compared by the Kruskall Wallis test. Receiver operator characteristic (ROC) curves were constructed to evaluate the potential of antibodies to discriminate between animals with high or low/undetectable PVL. In this analysis, the level of *in vivo* infection or PVL was considered as the reference status and only two categories were considered: high and undetectable/low. Sensitivity was defined as the proportion of animals with high PVL which were correctly identified, whereas specificity was defined as the proportion of animals with undetectable/low PVL, compatible with the aleukemic stage, which were correctly identified. The Spearman rank test was used to analyze the correlation between PVL and p24 antibody levels. For all the analyses, a value of *P* < 0.05 was considered significant. All data were analyzed with GraphPad Prism for Windows v. 5.01. MedCalc v. 12.3.0.0 was used to compare areas under the ROC curve statistically.

## Results

**Figure 1:**
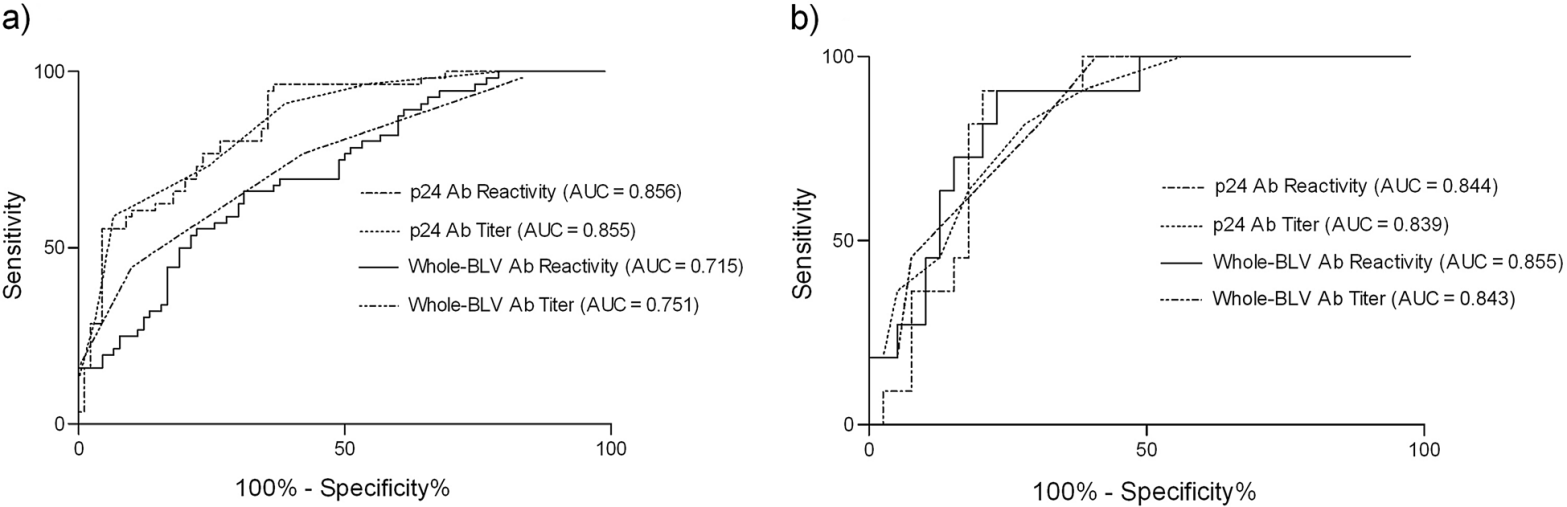
**ROC curve analysis for BLV antibody reactivities and titers to predict the blood PVL in the two herds under study: a) 51, b) T1.** For this analysis, the PVL was divided into two groups: high and low/undetectable. Ab) antibody.

**Figure 2:**
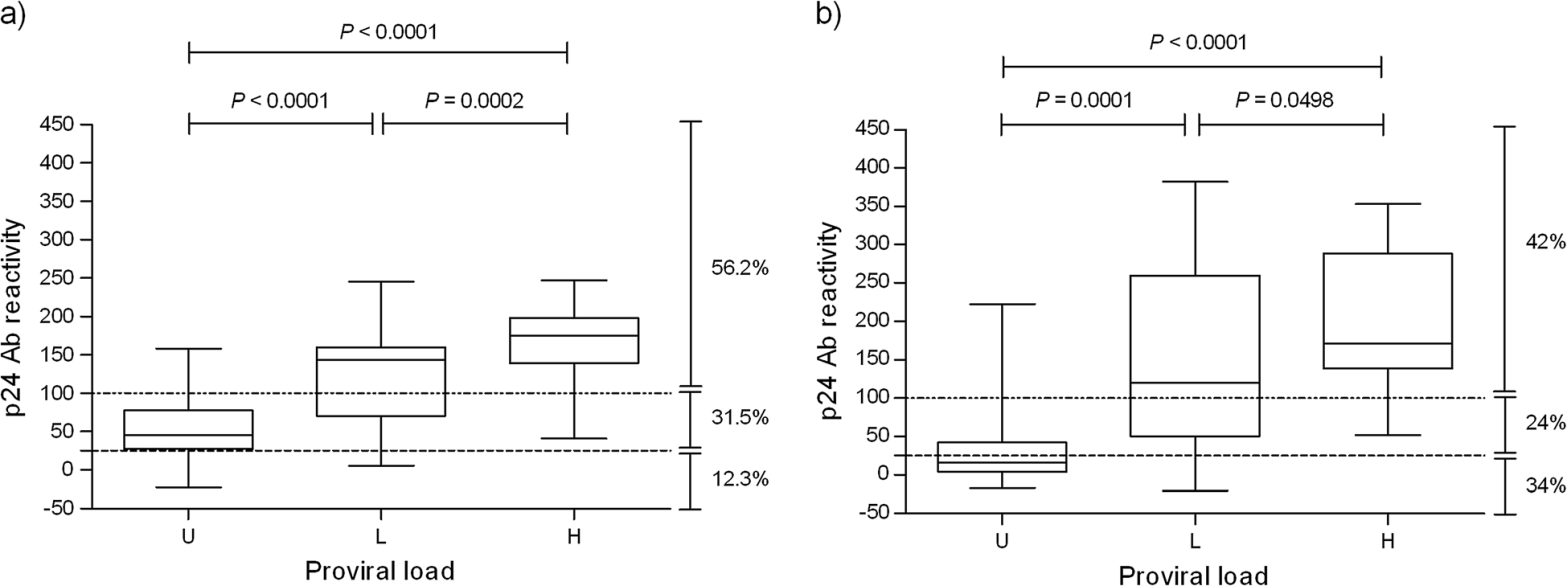
**p24 antibody reactivity descriptive analysis in different PVL categories.** Box and whisker plots show p24 antibody reactivity of the cows depending on their PVL category in herds: **a**) 51 and **b**) T1. U) undectectable, L) low and H) high proviral load, Ab) antibody. The Kruskal-Wallis test was used to evaluate differences between categories, *P* values are shown.

The prevalence of p24 antibodies on the 15 farms studied was over 66% (Table 1). The prevalence of whole-BLV antibodies and PVL quantification were analyzed in all the samples (n = 196) taken from herds T1 and 51 (Table 2). In both herds, whole-BLV antibodies were detected in a greater proportion of animals than p24 antibodies (Table 2). More than 20% of the p24 seroreactors showed undetectable PVL and more than 20% showed low PVL (Table 2), being both categories compatible with the aleukemic infection stage. The rate of animals with high PVL among p24 seroreactors and hence, with more than 1% BLV-infected PBMCs, was 33.3% on farm T1 and 43.8% on farm 51. All the animals stated as positive to whole-BLV antibodies and negative to p24 antibodies from both farms (n = 21) showed undetectable (20/21) or low (1/21) PVL (data not shown). ROC analysis showed a higher AUC for p24 antibodies than whole-BLV antibodies (Z_reactivity_: 3.55, *P* < 0.001; Z_titer_: 2.88, *P* < 0.01), and as consequence a better performance to predict the proviral load status in herd 51 (Figure 1a). No significant differences were found between the performance of p24 and whole-BLV antibodies in herd T1 (Z_reactivity_: 0.26, *P =* 0.79; Z_titer_: 0.09, *P* = 0.93) (Figure 1b). Both, the p24 reactivity and titer showed similar AUC values and resulted in a similar estimation of sensitivity and specificity (Figure 1). The p24-antibody reactivity from animals with high PVL was significantly higher than those from animals with low and undetectable PVL (Figure 2) in both herds.

**Table 2:** Prevalence of antibodies and proviral load

**Herd**	**p24 antibodies pos**	**whole-BLV antibodies pos**	**p24 seroreactors**		
			**PVL**		
			**U**	**L**	**H**
T1	66.0	82.0	21.2	45.5	33.3
51	87.7	100.0	32.0	24.2	43.8

All numbers are percentages. pos) positive, *PVL*) proviral load, *U*) undetectable, *L*) low, *H*) high.

**Figure 3:**
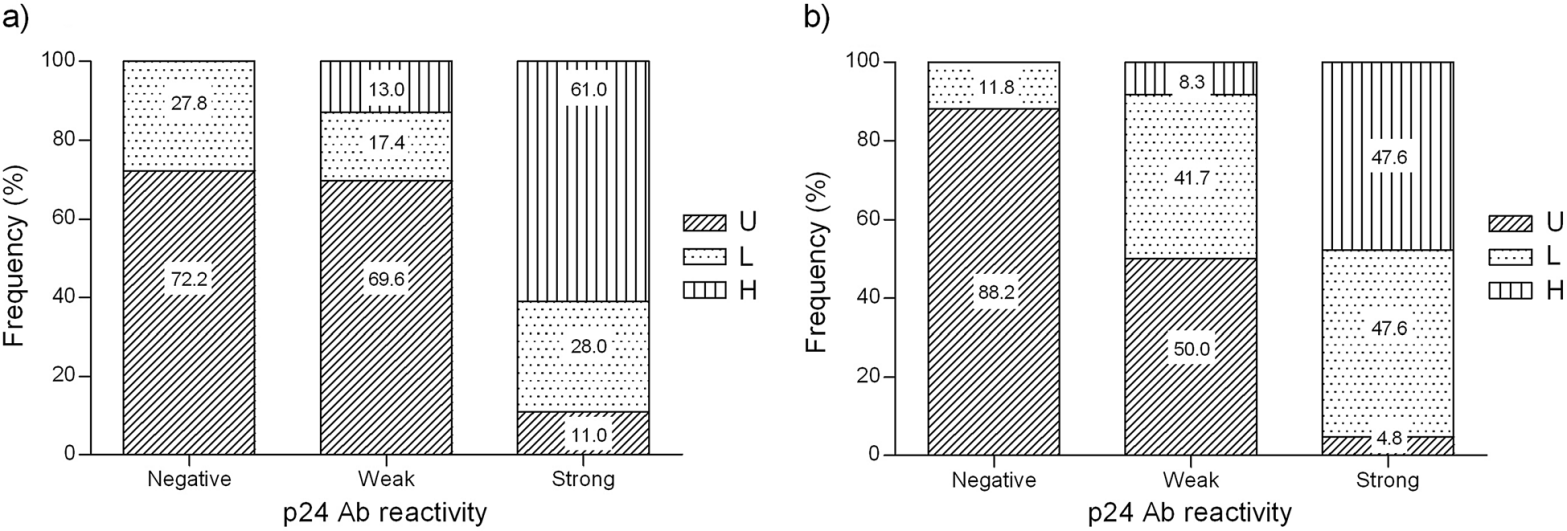
**Proviral load distribution in different p24 antibody categories.** The frequencies of animals with different levels of proviral load are shown for each p24 Ab category in herds: **a**) 51 and **b**) T1. U) undetectable, L) low and H) high proviral load, Ab) antibody

**Figure 4:**
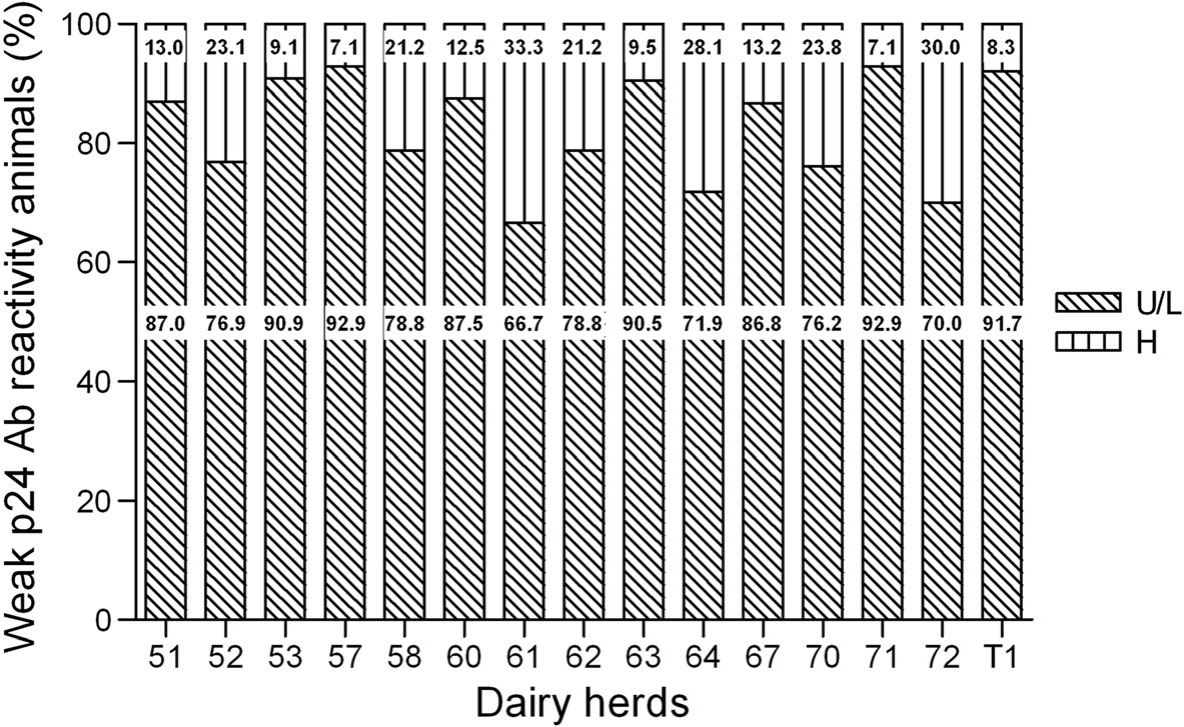
**Proviral load distribution in weak p24 antibody reactivity animals from 15 dairy herds. p24 seropositive animals were selected from 15 herds and analyzed for BLV proviral load.** The percentages of animals with undetectable/low (U/L) and high (H) proviral load are shown. Ab) antibody.

A significant positive correlation was observed between PVL values and p24 antibody reactivity in both farms (r _T1_ = 0.7, *P* < 0.001, r _51_ = 0.71, *P* < 0.0001), where the rate of animals with high PVL increased in concordance with the antibody category, and varied from 8.3 to 13% in weak reactors and from 47.6 to 61% in strong ones (Figure 3). According to the above findings that showed that a weak p24 antibody reactivity could be a good indicator of low PVL, the analysis was extended to the whole number of weak p24 antibody reactors (n = 311) of the other 13 farms, where 12.5% to 31.1% of all the seroreactors showed weak reactivity (Mean = 21.23, SD = 5.44) (Table 1). Figure 4 shows the distribution of PVL in the weak p24 antibody reactors from the different farms. The mean of high PVL reactors within this group was 17.38% (SD = 8.92). In 5/15 farms, the number of high PVL animals was lower than 10%. The highest proportion of high PVL animals was 33.3% on Farm 61, which showed 22.9% of the animals within the weak p24 subgroup (Table 1).

## Discussion

In this study, we analyzed the PVL and the antibody level of naturally BLV-infected cattle from fifteen different herds. We found that the humoral response reflected the level of *in vivo* infection, and may therefore have useful epidemiological applications.

We worked with 15 commercial herds, which are typical large dairy herds of the country considering productive parameters and BLV prevalence (Table 1). This study complements an ongoing work, in which the same animals are being studied for the presence of polymorphisms in the complete genome that could be associated with the BLV PVL outcome.

In all farms, we found animals with different PVL status (Figure 4), which confirmed the concept that BLV PVL varies between naturally infected animals, as previously reported in two previous works, both based on one farm case [3,4].

The results presented here provide a baseline to design an alternative control strategy based on the permanence of animals with low levels of infection in the herd, following the hypothesis that the level of proviral BLV in blood should play a major role in the success or failure of BLV transmission, as reported for experimental BLV infections [5,10] and natural Human T Lymphotropic Virus-1 (HTLV-1) infections [11]. The main goal of this program would be the rational control of intentional virus propagation, with the aim to obtain a low PVL in the whole herd, to finally diminish the risk of animal-to-animal transmission.

Under a practical point of view, the elimination of all infected animals with high proviral load is not be feasible on individual farms, since apart from mortality due to sporadic cases of lymphosarcoma, productive parameters are considered as normal in this group of cattle. In this context, the application of a selective segregation plan based on proviral load could be appropriate in a consortium-based strategy, where a group of farms work together toward the elimination of infection. In this case, animals with low proviral load should be recruited to form a low-transmission farm. In this scenario, the quantification using real-time PCR in whole natural herds to select animals that would be recruited to form the low-transmission farm would be extremely expensive. Hence, an alternative approach to select and recruit clean and low-infected animals becomes necessary. With this concept in mind, we analyzed the relationship between the individual level of infection and the serological profile with the aim to find an affordable indicator of PVL that could be used in local laboratories without the need of expensive equipment and reagents. We first analyzed whole-BLV and p24 antibodies in two herds, in which we quantified PVL in the total number of samples, with the aim to detect the best PVL predictor among the serological options. The ROC analysis showed that p24 antibodies allow predicting the PVL with a similar performance when titer or reactivity values are used for calculation (Figure 1). Even when whole-BLV antibodies were detected in a greater proportion of animals, we found no evident reason to work with these antibodies; as all these animals showed undetectable or low PVL. In our situation, this is important for two reasons. First, although the two ELISA antigens used were in-house, the p24-ELISA uses an *Escherichia coli* recombinant-derived antigen, which is less expensive and laborious to elaborate than the cell-derived antigen used in the whole-BLV ELISA. Secondly, there is no need to titrate the samples, since the strength of ELISA reactions seems to be as good as titer to predict the PVL (Figure 1). These two reasons make the serological analysis extremely cheap and straightforward. The prediction made by ROC analysis in these two farms was supported by the analysis of variance (Figure 2) and the correlation analysis, which showed that PVL is well reflected by the p24 antibody level.

Since the analysis of p24 antibodies showed a subpopulation of weak-p24 reactors in all herds (Table 1), we extended the analysis of PVL to this subgroup. We confirmed that a great proportion of animals of this subgroup showed undetectable or low PVL (Figure 3). This finding allows us to consider this subpopulation as a good candidate to be recruited as potentially low disseminators of infection. In a consortium-based approach, at least 20% of the animals of each farm should be considered as putative donors to form a BLV low-dissemination herd according to our analysis (Table 1).

Then, the control strategy would consist in the recruitment of animals with low or undetectable PVL using p24 serology as a low-cost method, instead of PVL quantification. This plan should be rationally designed considering that (1) all the animals that participate in the plan should be checked for p24 antibody reactivity, (2) negative and weak p24 reactors should be selected and recruited to form a new potentially low-transmission farm, (3) PVL should be checked up in the recruited group, in which animals with high PVL will still be present and should be eliminated, and (4) monitoring should be done in a regular basis to constantly “clean” high PVL individuals that might appear, even when PVL is thought to keep constant [12]. With this strategy, the transmission rate should become lower and lower as the animals with high propagation potential are removed.

A risk assessment should be made to analyze the dynamics of infection under these conditions, especially considering the theoretical extinction time when compared with naturally infected farms, as previously discussed [13].

Although this kind of control program based on the *in vivo* level of infection has been proposed [4], there are no reports showing the application and/or success of this strategy. In this context, a field trial will be designed and set up to analyze the feasibility and risk of the strategy, as a rational alternative to control BLV infection.

Finally, whether or not the immune response only reflects or also controls PVL is still unknown. Further studies should be carried out to define the reasons of the individual PVL differences and whether they are caused by the host genetic background, the viral strain, the infectious dose, and/or are due to multifactorial reasons. In the context of HTLV infection, the PVL is also correlated with the level of circulating antibodies [14,15] and the specific CD8+ lymphocyte response contributes to the control of the PVL and is associated with a lower risk of clinical disease progression [16]. Similar phenomena could be occurring with BLV natural infection and studies regarding the BLV-specific cytotoxic response and its relationship with the PVL and the humoral antibody response would be appropriate.

## Conclusion

Our investigation represents the first study showing that the level of BLV-specific antibodies reflects the circulating proviral load. Whereas the quantitative evaluation of blood proviral load using real-time PCR is expensive and technically demanding, the measurement of antibodies in blood by ELISA is relatively straightforward and could therefore constitute a cost-effective tool in a BLV control intervention strategy, especially in highly infected herds such as Argentinean dairy ones.

## Abbreviations

Ab: Antibody; AUC: Area under the curve; BLV: Bovine leukemia virus; FLK: Fetal lamb kidney; H: High; HTLV: Human T Lymphotropic Virus; L: Low; PBMCs: Peripheral blood mononuclear cells; PVL: Proviral load; ROC: Receiver operator characteristic; SD: Standard deviation; U: Undetectable.

## Competing interests

The authors declare that they have no competing interests.

## Authors' contribution

GG carried out assays, analyzed data and drafted the manuscript, HC did all the real-time PCRs of weak p24 Ab reactivity samples and provided part of the samples, IA helped doing real-time PCRs, CM, NP, RP, GM and ML prepared the samples, helped with ELISAs and real-time PCR, NF helped with statistical analysis, MP provided part of the samples, KT planned the study, performed statistical analysis and drafted the manuscript. All authors read and approved the final manuscript.
